# Do Matrix Metalloproteases and Tissue Inhibitors of Metalloproteases in Tenocytes of the Rotator Cuff Differ with Varying Donor Characteristics?

**DOI:** 10.3390/ijms160613141

**Published:** 2015-06-09

**Authors:** Franka Klatte-Schulz, Thomas Aleyt, Stephan Pauly, Sven Geißler, Christian Gerhardt, Markus Scheibel, Britt Wildemann

**Affiliations:** 1 Julius Wolff Institute, Center for Musculoskeletal Surgery, Charité-Universitaetsmedizin Berlin, 13353 Berlin, Germany; E-Mails: aleyt.thomas@gmx.de (T.A.); stephan.pauly@charite.de (S.P.); sven.geissler@charite.de (S.G.); christian.gerhardt@charite.de (C.G.); markus.scheibel@charite.de (M.S.); britt.wildemann@charite.de (B.W.); 2 Berlin-Brandenburg Center for Regenerative Therapies, Charité-Universitaetsmedizin Berlin, 13353 Berlin, Germany

**Keywords:** tenocytes, rotator cuff, age, degeneration, MMPs, TIMPs

## Abstract

An imbalance between matrix metalloproteases (MMPs) and the tissue inhibitors of metalloproteases (TIMPs) may have a negative impact on the healing of rotator cuff tears. The aim of the project was to assess a possible relationship between clinical and radiographic characteristics of patients such as the age, sex, as well as the degenerative status of the tendon and the MMPs and TIMPs in their tenocyte-like cells (TLCs). TLCs were isolated from ruptured supraspinatus tendons and quantitative Real-Time PCR and ELISA was performed to analyze the expression and secretion of MMPs and TIMPs. In the present study, MMPs, mostly gelatinases and collagenases such as MMP-2, -9 and -13 showed an increased expression and protein secretion in TLCs of donors with higher age or degenerative status of the tendon. Furthermore, the expression and secretion of TIMP-1, -2 and -3 was enhanced with age, muscle fatty infiltration and tear size. The interaction between MMPs and TIMPs is a complex process, since TIMPs are not only inhibitors, but also activators of MMPs. This study shows that MMPs and TIMPs might play an important role in degenerative tendon pathologies.

## 1. Introduction

Matrix metalloproteases (MMPs) are a large family of proteolytic enzymes, which can degrade all components of the extracellular tendon matrix [1,2]. Their activities are antagonized by the interaction with the tissue inhibitors of metalloproteases (TIMPs). The balance between MMPs and TIMPs plays a critical role in tendon degeneration and healing [3,4]. The healing after rotator cuff (RC) reconstruction is associated with high failure rates [5,6,7], mainly linked to the formation of inferior, disorganized scar tissue at the tendon bone insertion site [8,9]. The mechanisms underlying the poor tendon healing are widely unknown. Since the MMPs and TIMPs regulate tendon modeling and remodeling, it is hypothesized that the development of tendon pathologies is dependent on the MMP/TIMP balance [2,10].

MMPs are classified according to their substrate specificity, sequence similarity and domain organization into the six groups [11]. Collagenases like MMP-1, MMP-8, MMP-13, and MMP-18 primarily degrade the most important component of the tendon matrix, the collagens [12,13]. The gelatinases MMP-2 and MMP-9 mainly degrade smaller collagen fragments and gelatin. The stromelysins MMP-3, MMP-10, and MMP-11; the matrilysins MMP-7 and MMP-26; and the metalloelastase (MMP-12) primarily degrade glycoproteins and proteoglycans [1,13]. The group of membrane bound MMPs (including MMP-14 and MMP-17) has a regulatory function and can activate other MMPs [2,14].

The activity of MMPs is highly regulated by four endogenous antagonists (TIMP-1 to TIMP-4), which can inhibit all MMPs by the formation of stoichiometric complexes through a non-covalent interaction with a zinc-binding site in the MMPs. TIMPs are not specific to any single MMP group and their inhibitory effects are overlapping [15]. Notably, TIMPs do not only inhibit the active forms of MMPs, but also interfere with the latent form and regulate their activation process. Thus, TIMPs can have promoting as well as inhibitory effects on the regulation of cell growth, invasion, differentiation, apoptosis and angiogenesis [16,17].

The aim of the project was to examine the expression and secretion of MMPs and TIMPs in tenocyte-like cells (TLCs) of Supraspinatus (SSP) tendon tears from donors differing in their degenerative status, age and sex. We hypothesize that the donor characteristics influence the MMP and TIMP expression and secretion in the TLCs, which might lead to an imbalanced MMP/TIMP ratio in the donor risk groups.

## 2. Results and Discussions

### 2.1. Correlation of Donor Characteristics

First, correlations between the individual parameters of the radiographic examination were investigated. Strong correlations were observed between muscle fatty infiltration (MFI) and tendon retraction (r_s_ = 0.769, *p* < 0.001) and tear size (r_s_ = 0.607, *p* < 0.001) as well as between tendon retraction and tear size (r_s_ = 0.690, *p* < 0.001). Subsequently, correlations between radiographic parameters and age were evaluated. The MFI showed strong correlation with the age (r_s_ = 0.673, *p* < 0.001), whereas mild age-dependent associations were observed for tear size (r_s_ = 0.463, *p* = 0.011) and tendon retraction (r_s_ = 0.411, *p* = 0.024).

### 2.2. Average MMP and TIMP Expression and Protein Secretion over All Samples (n = 30)

**Figure 1:**
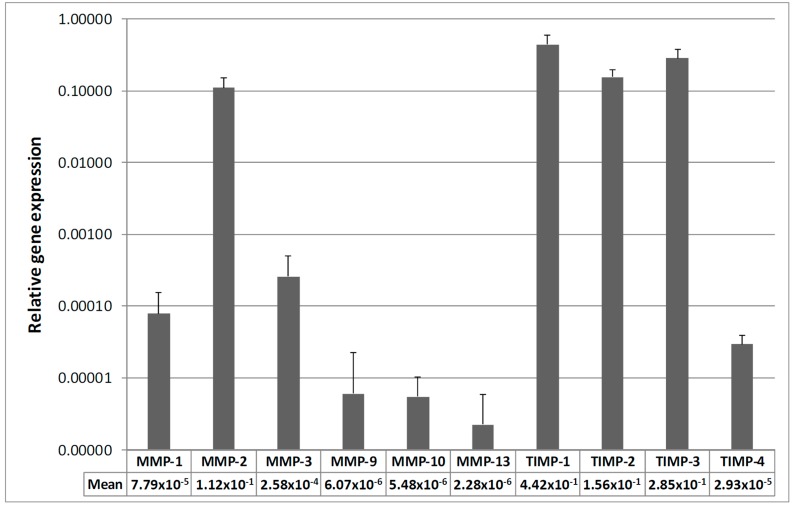
MMP and TIMP mRNA expression of all samples. Quantitative Real-Time PCR (qRT-PCR) analysis from torn RC tendons. The data represent the relative gene expression with 18S as reference gene using the Δ*C*_t_ method with efficiency correction. Data are expressed as the mean ± SD (*n* = 30) given in logarithmic form. MMP-2, TIMP-1, -2, and -3 showed the highest expression levels, while MMP-9, -10, and -13 showed lowest expression.

**Figure 2:**
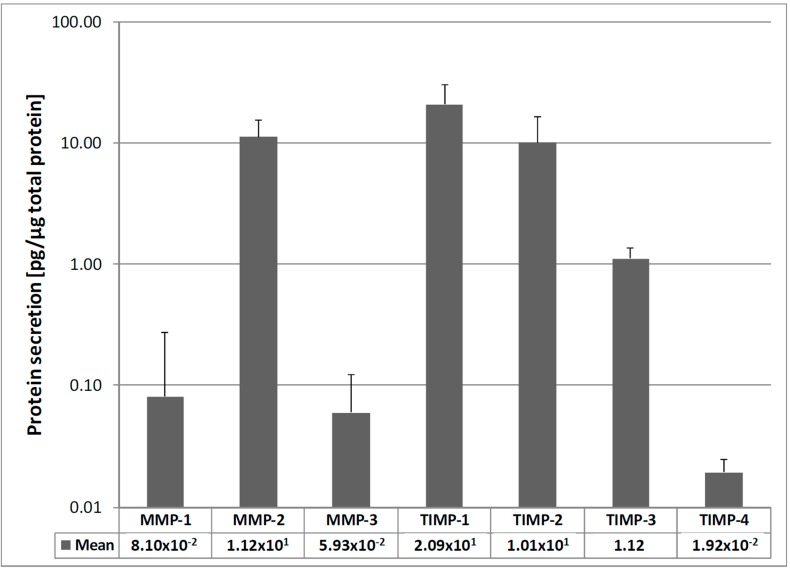
MMP and TIMP protein secretion of all samples. MMP-2 and TIMP-1 data were derived from sandwich ELISA. All other proteins were analyzed using Multiplex ELISA technique. All values were normalized to the total protein content (Coomassie Plus assay), given as mean ± SD (*n* = 30) represented in a logarithmic graph. MMP-2, TIMP-1, and -2 protein secretion was strongest in the cells, while MMP-1, -3, and TIMP-4 protein secretion was lower.

Gene expression analysis revealed MMP-2 expression to be the strongest in all TLCs, followed by MMP-3 and MMP-1. MMP-9, -10, and -13 were expressed in very low amounts, whereas MMP-10 and MMP-13 were only expressed in 26 or 28 samples, respectively. High expression levels of TIMP-1, -2 and -3 were found in all cells, while the TIMP-4 mRNA expression was much weaker (Figure 1). Due to the weak expression of MMP-9, -10 and -13, these MMPs were not analyzed on protein level. For the rest of the MMPs and TIMPs, the protein analysis of cell culture supernatants revealed a comparable pattern, where MMP-2 was the most secreted MMP and TIMP-1 and -2 the most secreted TIMPs in the cells (Figure 2). The FCS containing medium, which served as negative control, did not show detectable levels of MMPs or TIMPs in any of the ELISA analysis.

### 2.3. Relationship between Donor Characteristics and MMP/TIMP Expression and Secretion

Results of MMP/TIMP expression at mRNA and protein level did not differ significantly between TLCs of male and female donors. Therefore, all 30 TLC cultures were analyzed without separation regarding the donor sex.

**Figure 3:**
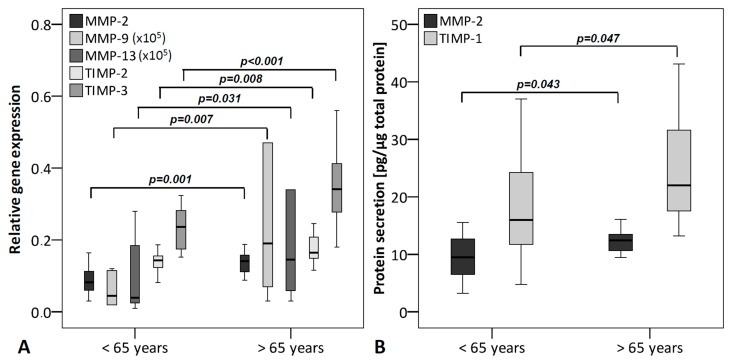
MMPs and TIMPs grouped according to the age of the donors (under 65 years (*n* = 16) and over 65 years (*n* = 14)). (**A**) qRT-PCR was performed to analyze gene expression. The box plot data represent the relative gene expression with 18S as reference gene using the Δ*C*_t_ method with efficiency correction. The mRNA levels of MMP-2, -9, -13 and TIMP-2, -3 are significantly increased with higher age; (**B**) Protein levels were analyzed using ELISA and normalized to total protein content (Coomassie Plus assay). Protein levels of MMP-2 and TIMP-1 were significantly elevated with higher age. To allow visualization of all MMPs/TIMPs in one figure, MMP-9 and -13 values were multiplied by 10^5^.

To determine the influence of donor age, donors were segregated into two groups: under 65 years (*n* = 16) and over 65 years (*n* = 14). The analysis revealed an age-dependent increase in the mRNA-expression levels of MMP-2, -9, -13 and TIMP-2, -3 (Figure 3A). This could only be confirmed at the protein level for MMP-2. In addition, protein levels of TIMP-1 were significantly elevated in TLCs from older donors, while mRNA expression was unaltered (Figure 3B). Spearman’s rho correlation revealed mild correlations between the age and the mRNA levels of MMP-2 (Spearman’s rank correlation coefficient (r_s_) = 0.504; *p* = 0.005), TIMP-2 (r_s_ = 0.485; *p* = 0.007), and TIMP-3 (r_s_ = 0.455; *p* = 0.012) (Table 1).

**Table 1:** Correlations between donor characteristics and MMPs and TIMPs.

Items		Relative Gene Expression						Protein Secretion		
		*MMP-2*	*MMP-9*	*MMP-10*	*MMP-13*	*TIMP-2*	*TIMP-3*	MMP-1	TIMP-1	TIMP-2
Age	r_s_	0.504	-	-	-	0.485	0.455	-	-	-
	*p*-value	0.005				0.007	0.012			
MFI	r_s_	-	0.432	-	-	-	-	-	0.413	-
	*p*-value		0.017						0.023	
Tendon retraction	r_s_	-	-	-	-	-	-	-	-	0.407
	*p*-value									0.029
Tear size	r_s_	-	0.451	−0.451	0.460	-	-	0.416	0.526	0.413
	*p*-value		0.012	0.012	0.010			0.022	0.003	0.023

**Figure 4:**
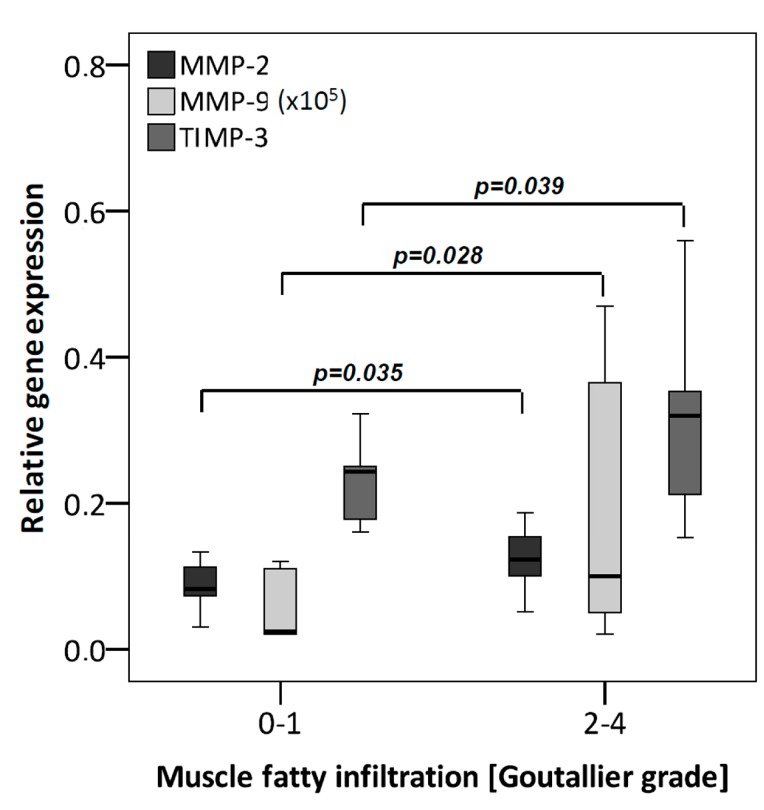
MMPs and TIMPs grouped according to the MFI (low MFI: Goutallier score 0–1 (*n* = 10); high MFI: Goutallier score 2–4 (*n* = 20)). qRT-PCR was performed to analyze gene expression. The box plot data represent the relative gene expression with 18S as reference gene using the Δ*C*_t_ method with efficiency correction. In the high MFI group increasing gene expression for MMP-2, -9 and TIMP-3 occurred. To allow a visualization of all MMPs/TIMPs in one figure, MMP-9 values were multiplied by 10^5^.

To analyze the association between MMP/TIMP expression and MFI, patients were grouped as follows: score 0–1 (*n* = 10) and score 2–4 (*n* = 20). The mRNA levels of MMP-2, -9 and TIMP-3 were significantly increased in TLCs from donors with enhanced MFI (Figure 4). At the protein level, none of the investigated proteins showed a significant alteration. MFI correlated mildly with mRNA values of MMP-9 (r_s_ = 0.432; *p* = 0.017) and protein values of TIMP-1 (r_s_ = 0.413; *p* = 0.023) (Table 1).

Tendon retraction was analyzed using two distinct groups: patients with the score of 0–1 (*n* = 9) *versus* patients with the score of 2–3 (*n* = 20). At mRNA and protein levels, no significant differences could be found between the two groups (data not shown). Nevertheless, tendon retraction showed mild correlation with protein secretion of TIMP-2 (r_s_ = 0.407; *p* = 0.029) (Table 1).

**Figure 5:**
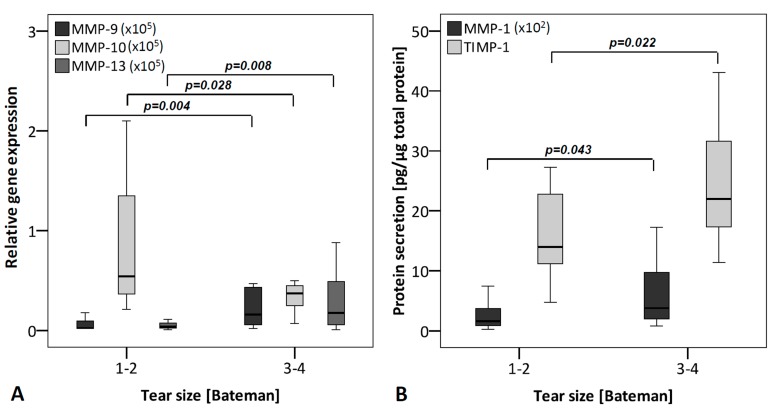
MMPs and TIMPs grouped according to the tear size (small tear size: Bateman score 1–2 (*n* = 12); big tear size: Bateman score 3–4 (*n* = 18)). (**A**) qRT-PCR was performed to analyze gene expression. The box plot data represent the relative gene expression with 18S as reference gene using the Δ*C*_t_ method with efficiency correction. MMP-9, and -13 expression of donors with bigger tear size was significantly increased, while MMP-10 expression was decreased at mRNA level; and (**B**) Protein levels were analyzed using ELISA and normalized to total protein content (Coomassie Plus assay). Protein level of MMP-1 and TIMP-1 showed an elevated secretion with bigger tear size. To allow a visualization of all MMPs/TIMPs in one figure, MMP-1 values were multiplied by 10^2^ and MMP-9, -10 and -13 by 10^5^.

Groups for analysis of the tear size were set as follows: score 1–2 (*n* = 12) and score 3–4 (*n* = 18). At mRNA level, MMP-9 and -13 were significantly enhanced in TLCs from donors with bigger tear size, whereas MMP-10 showed significantly decreased expression rates. At protein level, MMP-1 and TIMP-1 were significantly higher expressed with bigger tear size (Figure 5). A mild correlation was found between tear size and mRNA values of MMP-9 (r_s_ = 0.451; *p* = 0.012) and MMP-13 (r_s_ = 0.460; *p* = 0.010) and a mild negative correlation with MMP-10 (r_s_ = −0.451; *p* = 0.012). At protein level tear size correlated mildly with MMP-1 (r_s_ = 0.416; *p* = 0.022), TIMP-1 (r_s_ = 0.526; *p* = 0.003), and TIMP-2 (r_s_ = 0.413; *p* = 0.023) (Table 1).

### 2.4. Discussion

The healing of RC tears depends on the patient characteristic as well as the tendon tissue quality. However, the cellular or molecular mechanisms behind this relationship are still elusive. We previously showed that TLCs isolated from donors differing in age, sex and MFI exhibit different cellular characteristics and stimulation potential with BMP-2 and BMP-7 [18,19,20]. Since MMPs and TIMPs represent the main regulators of modeling and remodeling in the tendon, it was the aim to assess their expression and secretion in TLCs and their supernatant, related to the age, sex and degenerative status of the tendon. We hypothesized a change of the expression and secretion of MMPs and TIMPs in TLCs of risk groups, which might lead to an imbalance and therefore might explain the weaker healing potential in these patient groups.

In fact, we observed a predominately increased expression and secretion of MMPs and TIMPs in TLCs of older donors and of donors with higher degenerative status of the tendon.The most prominent changes within the MMPs were found for the gelatinases MMP-2 and MMP-9. Gelatinases such as MMP-2 and -9 mainly degrade smaller collagen fragments or already degraded collagen (gelatin) [21]. Since tears of the RC are mainly due to degeneration, this may indicate an important role of the gelatinases in later steps of the degeneration process after the full collagen structure was already degraded by other MMPs such as the collagenases. Within the TIMPs, mostly TIMP-1, -2 and -3 were regulated with donor characteristics.

In the present study, no difference in expression or secretion of MMPs and TIMPs were found between TLCs of male and female donors. Estrogen was reported to increase MMP-13 expression in rat Achilles tendon cells when directly applied to the cells [22]. Hormones such as Estrogen may not play a major role when they are not directly added to the cells. However, it has to be kept in mind that the FCS in the medium contains an undefined amount of hormones and the phenolred, which is present in the cell culture medium, can act as a weak Estrogen mimic [23]. Since tenocytes of male and female donors were shown to express Estrogen receptors [24], both components might influence the TLCs of male and female donors in the same manner.

The age of the donors is an important factor influencing the healing outcome after RC surgery and a cut off is mainly described at the age of 60 to 65 years [7,25]. In the present study, with an age cut off at 65 years, a significantly enhanced expression and secretion of MMP-2, -9 and -13 as well as TIMP-1, -2 and -3 was found and confirmed by correlation analysis. Results for MMP-2 and -9 are in accordance with Yu *et al*. who found the same changes in rat Achilles tenocytes [26]. In contrast to our findings, a decreased expression of TIMP-1 and TIMP-2 was reported. This is probably due to differences in experimental parameters such as donor species and age [26]. Similarly, another group reported increased MMP-1 activity in SSP tendons of elderly donors [10]. However, this was not observed in the present study.

The SSP tendon has a high collagen turnover, which is linked to high MMP activity levels in the tendon [10]. MMP-1, MMP-2, MMP-3, MMP-9, MMP-10 and MMP-13 are mainly described in the context of tendon healing. However, direct comparisons are critical, since most studies analyzed differences in MMP/TIMP expression between healthy and ruptured tendons using tissue sections and not cultured TLCs from ruptured RC tendons with different degeneration. MFI is a predictor for high degenerative status of tendons. Here, we found that enhanced MFI is correlated with significantly increased expression of MMP-2 and -9 in TLCs. A high MFI is the result of a barely moved shoulder due to the tendon rupture and insufficient mechanical stimulation of the tendon [27]. As mechanical stimulation was reported to influence MMP expression in rat tenocytes [28], this might explain possible alterations of MMPs with increasing MFI. A bigger tear size was found to be positively correlated with the increase in RNA levels of MMP-9 and -13 and protein level of MMP-1. These findings are in line with other studies, showing increased expression of these MMPs in ruptured Achilles or RC tendons compared to intact controls [10,29,30]. In contrast, Castagna *et al.* described no changes in protein levels of MMPs and TIMPs between SSP tendons of a torn area *versus* a macroscopically healthy area and the intact subscapularis tendon in 13 patients [31]. A recent study reports that the MMP-9 expression is associated with tendon retraction [30]. In contrast, others found a decreased gelatinolytic activity (MMP-2, MMP-9 and MMP-13) in ruptured SSP tendons compared to normal tendons [10]. MMP-3 was presently neither regulated on RNA nor protein level regarding the degenerative status of the tendon or other donor characteristics. Contradictory findings were observed by other authors, who reported that MMP-3 expression was mostly downregulated in ruptured or tendinopathic tendons [10,29,30]. It was hypothesized that the role of MMP-3 as an activator of other MMPs is important to bear a correct remodeling process [10].

In general, an increased MMP activity on the *in vivo* level could cause an imbalance between MMPs and TIMPs, which might lead to an excessive degradation of the tendon extracellular matrix and therefore to an inferior healing. It is clinically observed that the healing after RC reconstructions is often inferior in patients with higher age or degenerative status [25,27,32]. These clinical findings may be a result of increased activity of MMPs, as found presently. However, beyond the MMPs also TIMP-1, -2 and -3 were increased with higher age, MFI, and tear size. An increased TIMP-1 expression in the healing SSP tendon of rats was also described by Choi *et al.* [33]. Additionally, an elevated TIMP-1 level in plasma samples was found in patients with full thickness tears of the RC [34]. Other studies described that TIMP-2, TIMP-3 and TIMP-4 expression was decreased in ruptured Achilles or RC tendons compared to intact controls [29,35]. The presently observed increased TIMP expression and secretion might be a direct response of the cells to prevent an elevated MMP activity in their microenvironment. However, it is not clear if tendon rupture or degenerative changes are either cause or consequence of altered MMP/TIMP expression and activity levels. It was described that a failed healing investigated more than six months after RC reconstructions correlated with an increased MMP-1 and MMP-9 expression analyzed at the time of surgery which indicates that the biological environment at the time of surgery may directly influence the healing outcome [36]. Additionally, TIMPs are not only inhibitors of MMPs, but are also able to regulate the activation of MMPs [14]. Furthermore, this might explain the increased amounts of TIMPs and underlines the complex process of interaction between MMPs and TIMPs.

With the knowledge that an imbalance between MMPs/TIMPs in a tissue may challenge its healing capacity, it can be hypothesized that the use of MMP inhibitors could improve tendon healing. This hypothesis was examined previously by injecting substance P, an inhibitor of endopeptidases, in operative Achilles tendon repair in rats, which resulted in improved biomechanical properties [37]. Other studies used the MMP inhibitors doxycycline and α-2-macroglobulin for the repair of SSP tendons in rats and found decreased collagen degradation and improved collagen organization and biomechanical competence of regenerated tissues [38,39]. However, broad-spectrum MMP inhibitors as therapeutic agent have to be used with caution. Since MMPs are involved in various other functions in the body, they might cause undesired side effects. The goal would therefore be to develop more selective MMP inhibitors [40].

### 2.5. Limitations of the Study

TLCs were cultured in medium containing 10% heat inactivated FCS. FCS contains growth factors as well as some MMPs, which might have influenced the MMP and TIMP expression and secretion in the TLCs. However, the FCS was heat inactivated, which reduces the growth factor concentration as well as MMPs, as described for human plasma samples [41]. Furthermore, all cells were cultured identically, which should have affected all cells in the same manner.

Culturing primary cells in 2D culture is always a critical point, due to the risk of differentiation. Analyzing RNA directly isolated from the tendon tissue would avoid this problem, but is very challenging because of the limited biopsy size and RNA available. Therefore, only cells at very low passages (passages 1 and 2) were used to minimize differentiation problems. Mazzocca *et al.* described that tenocytes cultured in 2D culture can be used within the first three passages until a phenotypic drift occurs [42].

In the present study, we were not able to fully ensure that pure tenocyte cultures were used for the experiments. It might be possible that the presence of immune cells such as macrophages or lymphocytes could have affected the results of the study, because they are also able to express MMPs and TIMPs. However, as we could show previously, the tenocyte cultures isolated with the same method were characterized by flow cytometry analysis and expressed hematopoietic markers (CD11b, CD14, CD19, CD34, CD45) in less than 2% [18,19,20]. Therefore, the presence of immune cells in the tenocyte cultures can be nearly ruled out.

Data regarding the baseline of MMP and TIMP expression in cells of intact tendons would be a helpful additional information to validate the findings of the present study. However, SSP samples from age-matched patients (43–76 years) will never be totally intact, as RC tendons often undergo degenerative changes through age, which might have an effect on the MMPs and TIMPs as well.

As it is understood that mechanical stimulation plays an important role in the MMP/TIMP expression, the static 2D cell culture applied in this study might have had an effect on the MMP and TIMP expression and secretion. Furthermore, knowing the movement history of the RC patients prior to surgery could improve our study. However, this is not possible, but it can be speculated from the MRI data of the SSP muscle that patients with a high MFI have a longer or more severe pain history with decreased movement. Additionally to that, it was not possible to examine the period of time when the RC tear initially occurred. This is reasonable, because RC tears are mostly of degenerative nature, which is a slow process. Even if the patients above 50 years of age had an injury, mostly a degenerative pathology preceded [43]. However, there might be a correlation between the time period and the MFI as well.

Within the present study, we concentrated on analyzing the most described MMPs and the TIMPs. However, other proteases also play a role in extracellular matrix remodeling and therefore tendon healing such as cathepsins, a desintegrin and metalloproteases (ADAMs) or their attendant subgroup a desintegrin and metalloprotease with trombospondin motifs (ADAMTs). Therefore, the present study can only give a limited insight into the mechanisms of tendon degeneration.

## 3. Experimental Section

### 3.1. Tendon Material

TLCs were isolated from SSP tendon biopsies from 16 male and 14 female donors undergoing arthroscopic or open shoulder surgery. Biopsies were obtained (3–5 mm) from the proximal edge of the torn tendon. All patients gave their written informed consent and the local ethics committee of the Charité-Universitaetsmedizin Berlin authorized the anonymous use of tendon samples, which would otherwise be discarded (Ethic number: EA1/060/09).

### 3.2. Evaluation of Clinical Data

For each patient, the sex, age, and radiographic scores such as MFI according to Goutallier *et al.* [44] (MRI-assessed [45]), tendon retraction according to Patte *et al.* [46], and tear size according to Bateman [47] were collected. Beyond this, retrospective patient identification was impossible. Donor characteristics are recorded in Table 2.

**Table 2:** Donor characteristics.

Number	Age	Sex (m-Male/f-Female)	Fatty Infiltration (Goutallier Grade)	Tendon Retraction (Patte Grade)	Tear Size (Bateman Grade)
1	43	m	0	1	3
2	46	m	0	0	2
3	50	m	1	1	1
4	54	m	0	-	2
5	55	m	0	1	3
6	58	m	1	2	4
7	61	m	3	3	4
8	62	m	2	2	2
9	62	m	3	2	2
10	67	m	2	2	3
11	68	m	4	3	3
12	69	m	3	3	4
13	69	m	4	3	4
14	70	m	2	2	2
15	73	m	3	2	2
16	73	m	2	2	3
17	48	f	1	1	2
18	52	f	0	1	1
19	53	f	0	2	2
20	60	f	3	3	4
21	61	f	1	1	2
22	62	f	3	2	3
23	63	f	2	2	3
24	69	f	4	2	4
25	70	f	2	0	3
26	71	f	3	3	4
27	71	f	2	1	2
28	74	f	2	2	3
29	75	f	4	2–3	4
30	76	f	3	3	4

### 3.3. Cell Isolation and Culture

TLCs were isolated by collagenase digestion as described previously. The previous characterization study revealed a distinct tenocyte phenotype for cells isolated using this method [48]. Cells were cultured with growth medium (DMEM/Ham’s F12 with 10% heat inactivated FCS and 1% Penicillin/Streptomycin, all Biochrom AG, Berlin, Germany) at 37 °C, with 95% humidity and 5% carbon dioxide, with a medium change three times per week. When TLCs reached a minimum of 5 × 10^5^ vital cells they were cryo preserved until use.

### 3.4. Gene Expression Analysis

Cells from each donor (passage 1 or 2) were seeded in three wells of a 6-well plate and cultured under standard conditions for 7 days until they grew 80%–90% confluent. RNA was isolated from the cells using the NucleoSpin RNA II Kit (Macherey Nagel, Düren, Germany) according to the manufacturer. RNA quantity and purity was analyzed with a Nanodrop ND-1000 Spectrophotometer (PeqLab Biotechnologie, Erlangen, Germany). A total of 100 ng RNA were transcribed into complementary DNA (cDNA) using the qScript cDNA Supermix (Quanta BioSciences, Gaithersburg, MD, USA) with the Epgradient Mastercycler (Eppendorf, Hamburg, Germany). As PCR template, 1.25 ng cDNA were used for the qRT-PCR analysis. The cDNA was diluted 1:3 with Sybr Green mastermix (Quanta BioSciences) containing 10 µM of forward and reverse primer mix to a total volume of 15.5 µL. All primer sequences were designed using Primer 3 software (Freeware; Available online: http://frodo.wi.mit.edu/primer3), and were produced by Tib Molbiol, Berlin, Germany (Primer sequences see Table 3). A qRT-PCR program with an initial denaturation step for 3 min at 94 °C was used, followed by an amplification program with 40 repeated cycles (95 °C for 15 s, 64.2 °C for 45 s, 72 °C for 30 s), and a melting curve program. For all primers, amplification efficiencies were analyzed and relative gene expression calculated using the Δ*C*_t_ method with efficiency correction. In previous studies 18S was found to be the most stable gene compared to other housekeeping genes in TLCs and was therefore used as reference gene. If no gene expression was measurable within amplification, the *C*_t_ value was set at 37, where only unspecific amplification or primer dimers can be expected.

**Table 3:** qRT-PCR Primer.

Gene	Accession No.	Primer Sequence	Size (bp)
*18S RNA*	NM_022551	Forward: 5′-CGGAAAATAGCCTTTGCCATC-3′	107
		Reverse: 5′-AGTTCTCCCGCCCTCTTGGT-3′	
*MMP-1*	NM_002421.3	Forward: 5′-CACGCCAGATTTGCCAAGAG-3′	148
		Reverse: 5′-GTCCCGATGATCTCCCCTGA-3′	
*MMP-2*	NM_004530	Forward: 5′-TGGATGATGCCTTTGCTCGT-3′	156
		Reverse: 5′-CCAGGAGTCCGTCCTTACCG-3′	
*MMP-3*	NM_002422.3	Forward: 5′-TGGGCCAGGGATTAATGGAG-3′	104
		Reverse: 5′-GGCCAATTTCATGAGCAGCA-3′	
*MMP-9*	NM_004994.2	Forward: 5′-GGGACGCAGACATCGTCATC-3′	150
		Reverse: 5′-GGGACCACAACTCGTCATCG-3′	
*MMP-10*	NM_002425	Forward: 5′-CCACCTGGACCTGGGCTTTA-3′	192
		Reverse: 5′-GAACTGGGCGAGCTCTGTGA-3′	
*MMP-13*	NM_002427.3	Forward: 5′-CCTTCCCAGTGGTGGTGATG-3′	144
		Reverse: 5′-CGGAGCCTCTCAGTCATGGA-3′	
*TIMP-1*	NM_003254.2	Forward: 5′-TTGGCTGTGAGGAATGCACA-3′	128
		Reverse: 5′-AAGGTGACGGGACTGGAAGC-3′	
*TIMP-2*	NM_003255.4	Forward: 5′-CCTGAGCACCACCCAGAAGA-3′	123
		Reverse: 5′-TCCATCCAGAGGCACTCGTC-3′	
*TIMP-3*	NM_000362.4	Forward: 5′-CCGAGGCTTCACCAAGATGC-3′	140
		Reverse: 5′-GCCATCATAGACGCGACCTG-3′	
*TIMP-4*	NM_003256.3	Forward: 5′-GAAGCCAACAGCCAGAAGCA-3′	120
		Reverse: 5′-TTCCCTCTGCACCAAGGACA-3′	

### 3.5. Multiplex ELISA

The protein concentration of the four TIMPs, MMP-1 and MMP-3 in the cell culture supernatant was analyzed using Magnetic Luminex Performance Assays (TIMP Multiplex Kit, MMP base kit plus MMP-1 and MMP-3 kit, R&D Systems, Abingdon, UK) according to the manufacturer. Briefly, cell culture supernatants from three 6-wells were pooled. Standards and pooled supernatants were measured in duplicates. Medium with 10% FCS served as negative control. Supernatants were diluted 1:4 for TIMP assay and used undiluted for MMP-1/MMP-3 assay. An automated magnetic washing device (Bio-Plex^®^ Pro II, BioRad Laboratories, Munich, Germany) was used. The assays were analyzed using the Bio-Plex^®^ 200 System (BioRad) with the Bio-Plex Manager software. Protein concentrations of the TIMPs and MMPs were normalized to the total protein content in the supernatant analyzed by Coomassie Plus assay (Thermo Fisher Scientific, Dreieich, Germany).

### 3.6. Sandwich ELISA

TIMP-1 concentration was further analyzed by conventional sandwich ELISA (TIMP-1 DuoSet ELISA, R&D Systems). Protein concentration of MMP-2 was analyzed using Total MMP-2 Quantikine ELISA (R&D Systems) according to the manufacturer instructions. Standards and pooled cell culture supernatants were measured in duplicates. Medium with 10% FCS served as negative control. Supernatants were diluted 1:4 for MMP-2 ELISA and 1:200 for TIMP-1 ELISA. Protein concentrations of TIMP-1 and MMP-2 were normalized to total protein content (Coomassie assay).

### 3.7. Statistics

Statistical analysis was performed using SPSS 20 (IBM, Armonk, NY, USA). For the analysis of significant differences between two cohorts, the radiographic scores were grouped as follows: the cut off for MFI and tendon retraction was defined between grade 1 and 2, and the cut off for tear size between grade 2 and 3. An age cut off was defined at 65 years in accordance with the literature [6,7]. Mann-Whitney U test was performed to analyze significant differences between the defined groups. The box plots represent the median with 25th to 75th percentile and the whiskers are placedat 1.5 times the interquartile range below/above the first/third quartile of the box. Spearman’s rho test was used for correlation analysis and is given as Spearman’s rank correlation coefficient (r_s_). Statistical dependence between two variables was only considered for correlation values above 0.4. A r_s_ of 0.4–0.6 was considered mild, whereas a r_s_ above 0.6 was considered as strong correlation. The level of significance was set at *p* < 0.05.

## 4. Conclusions

In the present *in vitro* study, TLCs from donors with higher age (>65 years) or degenerative status of the tendon showed increased mRNA and protein levels of mainly the gelatinases MMP-2 and MMP-9, but also the TIMPs were upregulated in theses donor groups. The interaction between MMPs and TIMPs is a complex process, since TIMPs are not only inhibitors of MMPs, but are also able to regulate the activation of MMPs. The results of the present study show that MMPs and TIMPs might play an important role in degenerative tendon pathologies, but also highlights the need of more knowledge in this research area.
